# A Method of Prophylaxis against Infestation with Lice

**Published:** 1918-01

**Authors:** 


### LOUSE PROBLEM

---

**A Method of Prophylaxis against Infestation with Lice.** Trans. of Abs. from the *Archives de Médecine et de Pharmacie Militaires,* May, 1916, p. 775, of an article by A. Rysell in the *Military Surgeon,* Vol. XXXVIII, No. 2, 1916, p. 238.

It is well known that laborers on the sulphurous lands of Sicily are immune to malaria. A common explanation of this fact is that mosquitoes are repelled by the odor of sulphur from secretions.

The writer has employed a precipitate of sulphur upon people from the orient infested with lice. He has found this a most effective destroyer of parasites.

The method of application is to spread two spoonfuls of powder with a brush on both sides of the shirt. A small quantity should also be spread on the seams of the sleeve and at the fork of the drawers. Lice are invariably killed by this procedure, and the author declares that it is equally effective against bed-bugs, fleas, flies, ticks, and other parasites.

Sulphur is preferable to naphthaline because it does not irritate the skin and because its odor is less pronounced.

In a complementary note the author declares, in reply to various queries, that his method is purely prophylactic and not curative. Precipitate of sulphur should be used, as the flowers of sulphur are ineffective for the purpose, and colloidal sulphur is too costly. Sulphur soap is ineffective, because the lice are on the clothing and not on the body itself.

Sulphur retains its power for at least two weeks, and sometimes for a month. Belts and neckties should be powdered as well as under-clothing.

